# Perceived Stress and Low-Back Pain Among Healthcare Workers: A Multi-Center Prospective Cohort Study

**DOI:** 10.3389/fpubh.2020.00297

**Published:** 2020-08-11

**Authors:** Jonas Vinstrup, Markus D. Jakobsen, Lars L. Andersen

**Affiliations:** National Research Centre for the Working Environment, Copenhagen, Denmark

**Keywords:** Cohen, psychological stress, pain, workplace, nurses, healthcare

## Abstract

**Objective:** This study aimed to investigate the association between perceived stress and odds of low-back pain (LBP) in a population of Danish healthcare workers.

**Methods:** Utilizing a prospective cohort design with 1-year follow-up, a total of 1,944 healthcare workers from 389 departments at 19 hospitals responded to questionnaires containing items related to lifestyle, health, and working environment. Using Cohen's Perceived Stress Scale, associations between baseline stress levels and LBP intensity (0–10 scale) at follow-up were modeled using cumulative logistic regression, accounting for clustering at the department level and adjusting for age, sex, baseline intensity of LBP, education, seniority, number of daily patient transfers, psychosocial work environment, and lifestyle factors.

**Results:** For the entire population, moderate and high stress (reference: low stress) at baseline increased the odds of LBP at 1-year follow-up with odds ratios (ORs) of 1.39 (95% CI 1.13–1.71) and 1.99 (95% CI 1.49–2.66), respectively. Sensitivity analyses among female nurses showed similar results [*i.e*., OR 1.40 (95% CI 1.08–1.80) and OR 2.08 (95% CI 1.44–3.00) for moderate and high stress, respectively], while only high stress significantly increased the odds among those without LBP at baseline.

**Conclusions:** Psychological stress increases the odds of LBP among healthcare workers. Identifying and diminishing work-related psychosocial stressors should be included in strategies that aim to prevent musculoskeletal disorders in this population.

## Introduction

The physiological phenomenon known as “stress” is—in animals and humans alike—most often characterized as the result of real or perceived threat to the organism, typically as a reaction to various external exposures (1). However, while the bi-directional stress processes between the brain and the cardiovascular, autonomic, and immune systems are essential in promoting short-term allosteric adaptations to stressors with the noble, primary goal of maintaining homeostasis, exposure to long-term stressful conditions leads to a wide array of negative health consequences (1, 2).

Indeed, persistent stress has been associated with numerous indicators of poor health, including abnormal cortisol patterns (3), depression and anxiety (4), type II diabetes (5), obesity (6), poor sleep (7, 8), persistent pain (9, 10) as well as cardiovascular- and all-cause mortality (11). Generally, greater lifetime stress severity predicts poor mental and physical health (2, 12, 13), and cumulative exposure to work-related stressors has been linked to absenteeism from work, increased healthcare costs, and decreased job performance (14–17). Following this, several work-related situational- and environmental stressors have been identified in the literature, including high job demands, poor support from colleagues, reward imbalance, job insecurity, and over-commitment (16, 18–21).

Specifically among healthcare workers, work-related stress has been associated with job (dis)satisfaction, burnout as well as poor mental- and physical health outcomes (22). Additionally, it is likely that certain job groups within the realm of healthcare are exposed to more work-related stressors than others: For example, a recent study among healthcare workers showed that nurses in primary care experience higher levels of perceived stress and lower levels of subjective well-being compared with their colleagues (23), indicating that this subgroup of the working population may exhibit an increased risk of negative health outcomes due to work-related stress. This notion needs to be considered in relation to the myriad of work-related factors known to influence pain among healthcare workers (24, 25), among which indicators of stress (*e.g*., emotional exhaustion, high emotional demands, and poor psychosocial safety climate) are known to significantly amplify the risk of musculoskeletal pain (24, 26).

However, while increases in cortisol levels have been shown to precede increases in pain levels hours later (27), it is currently unclear to what extent stress exacerbates future pain levels in healthcare workers. Therefore, the primary aim of this study was to investigate associations between baseline stress levels and odds of low-back pain (LBP) at 1-year follow-up in a population of Danish healthcare workers. Additionally, sensitivity analyses were performed, including individuals with and without LBP at baseline.

## Methods

Utilizing a prospective cohort design with baseline- and 1-year follow-up questionnaires, this study presents associations between stress levels and LBP intensity. Specifically, using Cohen's Perceived Stress Scale (28), we investigated associations between subjectively measured stress levels at baseline and odds of a one-point increase on the Visual Analog Scale at follow-up.

Prior to this analysis, we have previously published associations between lifestyle factors and the outcome of poor sleep and pain (8, 29), as well as data on the prospective association between patient transfers and back injury (30).

### Study Design and Participants

The baseline questionnaire was sent to 7,025 hospital workers from 389 departments at 19 hospitals in Denmark, of which 4,151 (59%) responded to the full questionnaire. Comparatively, Millar and Dillman (31) found an average of 42% response rate for web-based questionnaires, while Baruch (32)—based on 175 studies and more than 200,000 respondents—report a mean (SD) response rate of 55.6% (19.7).

In this analysis, we included healthcare workers who frequently engage with patients (*i.e*., nurses, healthcare assistants, nurses' aides, physical and occupational therapists, medical doctors, midwives, porters, and radiologists) and who responded to both baseline and follow-up questionnaires, yielding a final sample size of 1,944 healthcare workers. Furthermore, in subsequent sensitivity analyses, this study also includes associations specific to workers with and without LBP at baseline (*n* = 1,229 and *n* = 715, respectively), as well as to the subpopulation of nurses (*n* = 1,174), nurses without LBP (*n* = 413), and nurses with LBP at baseline (*n* = 761). Table 1 shows the baseline characteristics of the included participants.

**Table 1:** Demographics, work-, health-, and lifestyle variables at baseline.

	**All**			**All, with LBP**			**All, without LBP**			**Nurses**			**Nurses, with LBP**			**Nurses, without LBP**		
**Variable**	**Mean**	**SD**	**%**	**Mean**	**SD**	**%**	**Mean**	**SD**	**%**	**Mean**	**SD**	**%**	**Mean**	**SD**	**%**	**Mean**	**SD**	**%**
*N*	1,944			1,229			715			1,174			761			413		
Female			86.8			87.6			85.3			100			100			100
Age (y)	48.8	10.7		48.9	11.5		48.8	11.0		48.4	10.4		48.2	10.4		48.7	10.4	
BMI	25.2	4.6		25.4	4.6		24.8	4.4		25.2	4.7		25.4	4.7		24.8	4.6	
Smoking (yes)			8.4			8.2			8.3			8.0			8.2			7.5
Years in profession	18.3	11.6		18.3	11.5		18.3	11.6		18.6	11.4		18.6	11.6		18.5	11.0	
Frequency of daily patient transfers	3.5	2.2		3.7	2.2		3.2	2.0		3.8	2.0		4.0	2.1		3.4	2.0	
**Cohen's perceived stress**																		
Low stress (0–9)			32.1			25.0			44.2			32.4			25.2			45.5
Moderate stress (10–19)			56.8			61.8			48.3			56.9			61.6			48.2
High stress (20–40)			11.1			13.1			7.6			10.7			13.2			6.3
**Low-back pain within the previous 4 weeks (0–10)**																		
Low stress	1.6	2.2		3.2	2.2					1.6	2.2		3.3	2.2				
Moderate stress	2.5	2.5		3.7	2.3					2.5	2.5		3.6	2.3				
High stress	3.4	2.9		4.5	2.3					3.4	2.6		4.3	2.2				
**Leisure-time physical activity within the previous year**																		
Sedentary			6.7			7.4			5.7			5.4			5.7			5.1
Light exercise >3/week			62.6			64.5			58.3			64.9			66.8			60.3
Moderate exercise >3/week			27.7			26.1			30.9			27.9			26.3			31.5
Vigorous exercise several times per week			3.1			2.0			5.0			1.9			1.3			3.2
**Education**																		
Nurse			61.7			63.5			59.2			100			100			100
Doctor			11.7			9.5			15.2									
Healthcare assistant			7.0			7.2			6.8									
Physiotherapist			5.1			4.5			6.4									
Other			14.5			15.3			12.4									

### Outcome and Predictor Variables

The intensity of LBP was quantified by the following question:

*Rate your pain for the low-back within the previous 4 weeks (0–10, where 0 is no pain and 10 is worst imaginable pain)*.

Stress levels of the participants were quantified using Cohen's Perceived Stress Scale, the Danish version of which exhibits satisfactory validity, reliability, and internal consistency (33). It is a widely used instrument designed to measure “the degree to which individuals appraise situations in their lives as stressful” (28), and the scale shows well-established psychometric properties across populations (34, 35). Furthermore, it is often used as an outcome measure in intervention research (36, 37), allowing for the measurement of psychological stress among large cohorts. Presently, the 10-item questionnaire was used, with each item rated on a five-point Likert scale (ranging from “never” to “almost always”). The scores were subsequently summed, with higher scores indicative of higher levels of perceived stress. The normative values (mean ± SD) in the general population are 12.1 (5.9) and 13.7 (6.6) for men and women, respectively, with scores >20 considered as high (28). Therefore, a summed score between 0 and 9 was considered as “low,” 10–19 as “moderate,” and >20 as “high” in the present analysis.

### Covariates

In the “**Results**” section below, we report fully adjusted associations between baseline stress level and prospective odds of a one-point increase in LBP intensity. The analysis accounts for the following possible confounders relating to the individual, psychosocial, and working environment: Age, sex, LBP at baseline, education, body mass index, smoking, seniority, physical activity during leisure time, and frequency of patient transfer—as well as for factors related to the psychosocial work environment, *i.e*., recognition and influence at work.

### Ethics

By agreement with the Danish Data Protection Agency, the National Research Centre for the Working Environment is allowed to register all questionnaire studies in-house. According to Danish law, questionnaire- and register-based studies need neither informed consent nor approval from ethical and scientific committees. All data were de-identified and analyzed anonymously.

### Statistics

Associations between stress at baseline and LBP at follow-up were modeled using cumulative logistic regression (Proc Genmod, SAS), *i.e*., odds ratios (ORs) express the odds of LBP increasing one point on the 0–10 scale. Analyses were adjusted for the aforementioned covariates. To account for clustering, hospital department was entered in the “repeated subject” statement. Estimates are provided as ORs and 95% confidence intervals.

## Results

Among healthcare workers who were pain-free at baseline, we report LBP intensities of 0.65 (SD 1.57), 0.74 (SD 1.42), and 1.52 (SD 2.06) at 1-year follow-up for the low-, moderate-, and high-stress groups, respectively. For the sub-population of nurses who were pain-free at baseline, LBP intensities of 0.75 (SD 1.69), 0.74 (SD 1.44), and 1.5 (SD 1.9) for the three groups were reported at follow-up.

Furthermore, using “low stress” as reference, this study reports fully adjusted associations between stress levels and odds of an increase in LBP at follow-up among the full population of Danish healthcare workers (moderate stress: OR 1.44, 95% CI 1.12–1.86; high stress: OR 2.30, 95% CI 1.61–3.29). In the subgroup population who were pain-free at baseline, high stress—but not moderate stress—was significantly associated with increased odds (moderate stress: OR 1.25, 95% CI 0.89–1.76; high stress: OR 2.67, 95% CI 1.51–4.75). Likewise, in the sensitivity analysis including female nurses, high stress—but not moderate stress—was associated with increased odds at follow-up (moderate stress: OR 1.34, 95% CI 0.97–1.86; high stress: OR 3.17, 95% CI 1.88–5.37). Finally, in the subgroup of female nurses who were pain-free at baseline, high stress—but not moderate stress—was associated with increased odds of developing LBP (moderate stress: OR 1.09, 95% CI 0.71–1.69; high stress: OR 3.13, 95% CI 1.28–7.70) (Table 2).

**Table 2:** Stress level and odds of 1-point increase in low-back pain intensity (0–10) at 1-year follow-up.

	**All**			**All, without LBP at baseline**			**All, with LBP at baseline**		
**Stress score**	**OR**	**95% CI**	***p*-value**	**OR**	**95% CI**	***p*-value**	**OR**	**95% CI**	***p*-value**
Low (0–9)	1			1			1		
Moderate (10–19)	1.39	1.13–1.71	0.0016	1.25	0.89–1.76	0.1925	1.40	1.09–1.80	0.0094
High (20–40)	1.99	1.49–2.66	<0.0001	2.67	1.51–4.75	0.0008	1.89	1.33–2.70	0.0004
	**Nurses**			**Nurses, without LBP at baseline**			**Nurses, with LBP at baseline**		
**Stress score**	**OR**	**95% CI**	***p*****-value**	**OR**	**95% CI**	***p*****-value**	**OR**	**95% CI**	***p*****-value**
Low (0–9)	1			1			1		
Moderate (10–19)	1.40	1.08–1.80	0.0107	1.09	0.71–1.69	0.6848	1.46	1.08–1.96	0.0135
High (20–40)	2.08	1.44–3.00	<0.0001	3.13	1.28–7.70	0.0125	2.07	1.35–3.17	0.0008

*Values are presented as odds ratios and 95% confidence intervals*.

*Adjusted for age, sex, LBP at baseline, education, body mass index, smoking, seniority, physical activity during leisure time, frequency of patient transfer as well as recognition and influence at work*.

## Discussion

This study shows robust associations between perceived stress and increased odds of LBP among healthcare workers, utilizing Cohen's Perceived Stress Scale and the Visual Analog Scale, respectively. Specifically, for the whole population, a clear relationship was identified between stress levels (moderate and high) and increased odds of LBP at follow-up, while only high stress was associated with increased odds in the population free from LBP at baseline as well as for the subgroup of female nurses. This is important new knowledge in this occupational group, in which traditional preventive strategies have focused almost solely on ergonomic factors.

The main finding of this study—*i.e*., high stress being consistently associated with greater odds of future LBP—is in congruence with the existing literature on the relationship between psychosocial stress and pain (9, 10, 27, 38–40). For example, the population-based prospective cohort study by McBeth et al. (39) found that increased levels of evening-cortisol predicts the onset of persistent widespread pain at 15-months follow-up. Likewise, a Danish cross-sectional study including 4,739 adults reported strong associations between perceived stress and the use of over-the-counter analgesics (41). In the present cohort of healthcare workers, we find that baseline stress levels are highly predictive of increases in LBP intensity 1 year later, indicating that—among this and similar populations of the workforce—perceived stress constitutes a potent risk factor for developing future musculoskeletal disorders. Considering the biopsychosocial model of health by Engel (42), it is clear that individual perception of stress can be related to (and initiated by) an array of biomedical, psychological, and social stressors, presenting themselves in all shapes and sizes. For example, job demands, job control, over-commitment at the workplace, and social support from colleagues have been shown to predict stress outcomes (43), whereas emotional exhaustion was recently identified as a strong predictor of work-related injuries among healthcare workers (26). In relation to pain, the biopsychosocial model acknowledges potent influences such as catastrophizing, fear avoidance, expectation, meaning, context, *etc*.; all indicative of the substantial contribution arising from various psychosocial stressors (44). Following this, psychosocial stress in the workplace is traditionally gauged within the framework of the job *demand–control model* introduced by Karasek (45), describing work-related stress as an ongoing balancing act between strain/demands and control/support (45). However, in order to fully understand the dynamic bi-directional processes taking place during work-related stress (*i.e*., interactions between individual capacity and environmental stressors) as well as to shed light on the inconsistent relationships between various work-related stressors and performance, the *conservation of resources theory* (46) and the *challenge–hindrance model* (47) have since emerged. In combination, these frameworks emphasize the role of individual psychosocial factors (*e.g*., fluctuating psychological resources such as self-efficacy and the resulting difference as to whether a stressor is viewed as a challenge or hindrance), hereby further acknowledging the complexity of maneuvering a hierarchical, socially constructed workplace.

Collectively and despite historical disagreement on how to quantify stress, work-related stress is defined as “the general process in which individuals respond to and manage demands to meet multiple goals over time” (48). The results from the present study indicates that, regardless of the origin and nature of work-related stressors, high levels of perceived stress are strongly associated with increased odds of LBP, which—paradoxically—may very well turn into an additional stressor. This, in turn, initiates a rapacious circle in which the stressors and the stress response often become equally entangled and indistinguishable. This notion is echoed in the “**Results**” section, illustrating that participants experiencing pain at baseline are more like to suffer an increase in LBP due to moderate stress levels compared to their pain-free counterparts. Therefore, to counteract this, it appears critical that the ill-famed working environment in healthcare—known to cultivate high ratings of perceived exertion (49, 50), high prevalence of musculoskeletal disorders (25) as well as high levels of fatigue and stress (51)—undergo a substantial overhauling and reevaluation of core principles. The overarching aim of identifying and improving potent psychosocial influencers/stressors inherent to the local working environment should take precedence, as these issues arguably constitute the low-hanging fruits of this conundrum.

### Strengths and Limitations

Limitations of this study include the risk of underreporting of musculoskeletal disorders, recall- and non-response bias as well as ambiguity related to questions and questionnaire design; all inherent to questionnaire surveys (52–54). Additionally, although the vast majority of LBP cases are classified as “non-specific” (55, 56), the quantitative method used does not allow for the identification of possible contributors to pain in this population.

Strengths of this study include the adjustment for various confounders and inclusion of sensitivity analyses specific to nurses and pain-free participants as well as using Cohen's Perceived Stress Scale as a widely used and validated indicator of perceived stress. Finally, this analysis combines the advantages of a relatively large sample size, a prospective design as well as statistical adjustments for known physical- and psychosocial confounders. Future studies aiming to elaborate on these results should, if possible, include longer follow-up periods to confirm the robustness as well as to determine the generalizability of the presented results outside the hospital environment.

## Conclusion

In this cohort of Danish healthcare workers, perceived psychosocial stress was strongly associated with increased risk of LBP at 1-year follow-up, whereas being pain-free at baseline attenuated this relationship. Identifying and diminishing potent psychosocial stressors inherent to the local working environment are paramount in combating the high prevalence of musculoskeletal disorders seen in this population.

## Data Availability Statement

The datasets generated for this study are available on request to the corresponding author.

## Ethics Statement

Ethical review and approval was not required for the study on human participants in accordance with the local legislation and institutional requirements. Written informed consent for participation was not required for this study in accordance with the national legislation and the institutional requirements.

## Author Contributions

JV drafted the manuscript. MJ and LA revised it critically. All authors contributed substantially to the conception of the project as well as to the analysis and the interpretation of data, and provided approval for publication.

## Conflict of Interest

The authors declare that the research was conducted in the absence of any commercial or financial relationships that could be construed as a potential conflict of interest.
